# The multiagency approach to Sudden Unexpected Infant Deaths (SUID): eleven years’ experience in the Tuscany Region

**DOI:** 10.1186/s13052-020-00867-8

**Published:** 2020-07-20

**Authors:** Raffaele Piumelli, Niccolò Nassi, Annamaria Buccoliero, Rossella Occhini, Vincenzo Nardini, Paolo Toti, Cristina Salvatori, Marta Peruzzi, Cinzia Arzilli

**Affiliations:** 1 Sleep Breathing Disorders and SIDS Centre, Meyer Children’s Hospital, Florence, Italy; 2 Pathology Unit, Meyer Children’s Hospital, Florence, Italy; 3 Pathology Unit, S.Donato Hospital, Arezzo, Italy; 4 IInd Pathology Unit, University Hospital, Pisa, Italy; 5 Pathology Unit, University of Siena, Siena, Italy

**Keywords:** SIUD, SIDS, SUEND, Multiagency approach

## Abstract

**Background:**

The Sudden Unexpected Infant Death Syndrome (SUID) is one of the leading causes of mortality in the first year of life. The aim of this work was the retrospective evaluation of the incidence of SUID and the effectiveness of the multiagency approach to this phenomenon in the Tuscany Region.

**Methods:**

Data were obtained from the regional registry of SUID cases in the period 2009–2019. The registry contains both sudden unexpected deaths in the first week of life (Sudden Unexpected Early Neonatal Deaths - SUEND), and those occurring after the first week up to 1 year of age (SUID).

**Results:**

In this timeframe a total of 73 sudden unexpected deaths occurred in our region; 32 were Unexplained (i.e. Sudden Infant Death Syndrome - SIDS), 24 Explained, 10 Undetermined, and 7 SUEND. Autopsies were performed in 91% of cases, and in 95% of these by three groups of selected pathologists according to our protocol.

We found a low incidence of SUID (0.21 ‰), and SIDS deaths accounted for 0.1‰ of live births (48% of cases) with a high prevalence of infants of non-Italian ethnicity (38% of cases). Bereaved families were able to receive psychological support from mental health professionals and have contact with the family association, Seeds for SIDS. Audits were organized when post-mortem examinations were not carried out or carried out incorrectly in procedural terms, and when the diagnosis was particularly uncertain.

**Conclusions:**

This paper first provides data on SUID mortality based on complete post-mortems in an Italian region. According to these findings we can state that our approach is effective both in terms of correctly performed autopsies and support for bereaved families. Future efforts are necessary to further reduce the incidence of SUID especially among non- Italian infants. An improvement action is also recommended for ensuring a more accurate and consistent picture of the circumstances of death.

The final approval of the National Protocol for the management of SUID cases is therefore strongly advocated in order to improve surveillance in this specific field and abolish disparities among the Italian regions.

## Background

In an open-ended survey conducted in the USA by the American Academy of Pediatrics (AAP) Committee on Pediatric Research, the reduction of sudden infant deaths obtained with the “Back to Seep” campaigns was listed in second place among the seven great achievements in paediatrics over the last 40 years [1]. This emerged because Sudden Unexpected Infant Death (SUID) is one of the leading causes of mortality in the first year of life and have a devasting psychological impact on bereaved families. The Sudden Infant Death Syndrome (SIDS) is “the sudden death of an infant <1 year of age that remains unexplained despite an autopsy, review of the clinical history, and examination of the death scene” [2]. SIDS are part of SUID which encompass all sudden and unexpected deaths of natural and non-natural origin and of known and unknown causes. The timely and correct performing of autopsies in every SUID case represents a crucial point because it allows for precise monitoring of the phenomenon, providing an important contribution to research and a correct and empathetic communication of the results is highly supportive for bereaved families [3] [4].

A global approach to reduce the SUID phenomenon must include health policies aimed at promoting “Back to sleep Campaigns” for reducing the risk. Interestingly, risk factors for SIDS are the same as those identified for SUID, therefore the reduce the risk campaigns have been renamed “Safe to Sleep” in order to stress that SIDS reduction strategies are able to reduce all sleep-related deaths.

In Tuscany, a SIDS reduction campaign has been actively carried out by the Regional SIDS Centre of the Meyer Children’s Hospital, and the Seeds for SIDS family association in collaboration with the Tuscany Region Health department since 2002, whereas in 2009 a project aimed at implementing the multiagency management of SUID cases was also initiated. In this paper we report the data regarding SUID and discuss the effectiveness of our multiagency approach to this phenomenon in the Tuscany Region.

## Methods

In 2008, a multiagency group was created which included the regional healthcare department, the court, the regional SIDS Centre, the parents’ association ‘Seeds for SIDS’, a family paediatrician, a representative of the regional emergency departments, and three selected pathologists, one for each “vast area” into which the Tuscany Region is divided [5]. This task force established an algorithm for the management of SUID cases [6]. Our concerted action started on 1 January 2009 and was formally adopted in December 2009. The project aimed at implementing the correct management of SUID cases through an integrated organization working for the benefit of families in order to ensure sensitive investigations and identify the causes of death.

Each group of pathologists was responsible for the management of SUID cases in one of the three “vast areas” (North-West, South-East and Centre).

An intervention protocol, shared and approved by all members of the task force, establishes that when a SUID occurs, the emergency service (118) or the emergency department staff must fill in a form with the data related to the death scene (see Additional file 1). This is then submitted to the Regional SIDS Centre, the reference pathologists, and in the meantime the court is alerted.

The SIDS Centre Staff contact the families as soon as possible directly or through the family paediatrician. Bereaved families are invited to come to the SIDS Centre at the Meyer Children’s Hospital where a paediatrician collects information about the death circumstances, answers the parents’ questions about what happened and why, and provides information about SUID. A psychologist participates in these meetings to support the families and offer the possibility of planning a treatment program for grief management. A member of the Seeds for SIDS family association is also promptly available if requested.

In line with our procedure, the pathologist goes to the hospital where the infant has been transferred if the structure has the standard requirements for performing autopsies, otherwise the infant’s body is taken to the nearest hospital belonging to a list of previously identified reference autopsy centres. The three groups of pathologists involved in the project are always on call and a full post-mortem examination is carried out in accordance with the protocol.

The findings of the autopsy are disclosed to the families about 2 months later in a multiagency meeting with the pathologist, a paediatrician from the Regional SIDS Centre, a psychologist and a sub-specialist (i.e. cardiologist, geneticist, infectivologist, etc.) when required.

**Fig. 1:**
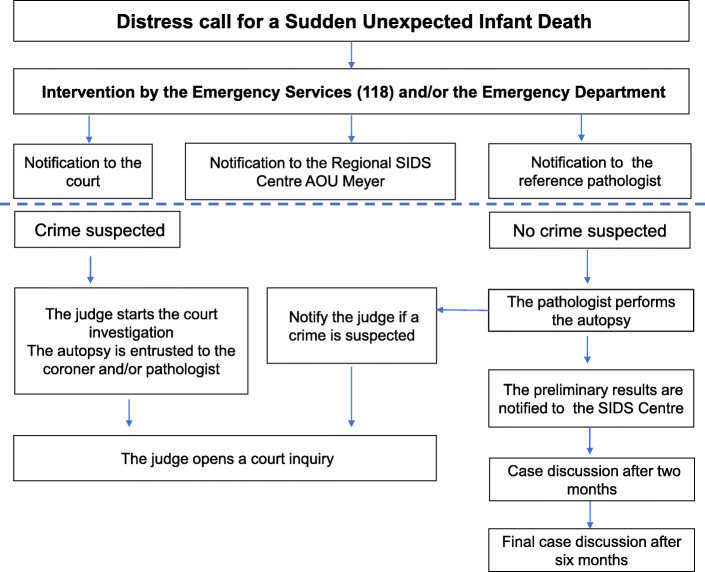
Multiagency approach to SUID. The flow-chart shows the procedure for the management of SUID cases

All the participants provide continued information and support for the family. A final case discussion meeting takes place 6 months later, when the final diagnosis is communicated (Fig. 1).

The dataset was obtained from the regional registry of SUID cases occurring in the Tuscany Region from 1 January 2009 to 31 December 2019. The registry, which is managed by the SIDS Centre Staff, records sudden unexpected deaths in the first week of life (Sudden Unexpected Early Neonatal Deaths - SUEND), and those occurring from the first week to the first year of life (SUID). SUEND were excluded from the statistical analysis due to their different pathophysiology and different risk factors [7].

Descriptive statistics are presented with numbers, percentages, medians, standard deviations and incidence.

## Results

In 11 years, there have been 66 SUID accounting for an overall mortality rate of 0.21 per 1000 live births, 32 of which were Unexplained (48%), 24 Explained (37%), and 10 Undetermined (15%) (Table 1).

**Table 1:** SUID and SUEND in the Tuscany Region

	2009	2010	2011	2012	2013	2014	2015	2016	2017	2018	2019	TOTAL
**SUEND**	0	2	0	0	0	2	0	1	0	1	1	**7**
**SUID Undetermined**	1	2	2	2	0	2	0	0	0	1	0	**10**
**SUID Unexplained**	4	4	1	5	1	0	4	4	3	4	2	**32**
**SUID Explained**												
**Suffocation, asphyxia**	0	0	0	0	0	1	1	0	0	0	0	**2**
**Natural Disease**	1	1	4	3	1	0	2	1	2	1	2	**18**
**Other letal event**	0	0	1	0	0	0	0	1	1	0	1	**4**
**Total**	**6**	**9**	**8**	**10**	**2**	**5**	**7**	**7**	**6**	**7**	**6**	**73**

*SUEND* Sudden Unexpected Early Neonatal Death

*SUID* Sudden Unexpected Infant Death

There were 7 cases of SUEND, all of which were subjected to autopsies performed by the same group of selected pathologists as recommended [8].

Of the 66 SUID, 43 were males (65%) and 23 (35%) females. Mean age was 3.2 months ±2.7. The geographic distribution was: 31 (47%) centre, 24 (36%) north-west and 11 (17%) south-east vast areas. Of these cases, 21 (32%) occurred in Autumn, 16 (24%) in Winter, 18 (27%) in Spring, and 11 (17%) in Summer. From the assessment of the ethnicity of the sample it was found that 38% of infants were of non-Italian parents.

SUID autopsies were performed in 91% of cases and among these, 95% were carried out according to our protocol. The mean time for performing the autopsy was 1.6 days ±1.6 standard deviations (range 0–8 days). The incorrect application of the protocol occurred in 9 cases (13.6%) because 6 autopsies were not performed due to lack of notification of the SUID case to our Centre and 3 were incorrect from a scientific point of view. In these 3 cases the judge decided to assign the autopsy to a coroner not belonging to our multidisciplinary group.

## Discussion

In Italy, the management of SUID should be regulated by the National Protocol which was lodged on October 7, 2014 [9]. This document contains the organisation plan, the list of professionals to involve in the event of SUID, and the description of the agreed autopsy protocol. It also illustrates the territorial distribution of the centres dedicated to performing autopsies and the multiagency approach to SUID cases. Unfortunately, this document has not yet come into force in Italy, therefore the management of SUID cases is highly variable. For this reason, in the Tuscany Region specific measures have been adopted as reported in the abovementioned decree [6].

Our data were obtained from the SUID case registry from 2009 to 2019. We found that gender, age of death, and seasonality were similar to those reported in a recent study carried out in Veneto, another Italian region [10], as well as those previously reported in other countries [11, 12]. The seasonality shows a prevalence in autumn-winter similar to the other Italian study but it differs from that reported in more recent studies which show a spring/summer prevalence [13], while a winter prevalence occurred before the reduce the risk campaigns [14]. Interestingly, in the entire SUID group, our SIDS rate was very close to that of the Veneto study (48% vs. 42%).

For the classification of the cases, infant death certificates were jointly discussed again among the SIDS Center staff and pathologists and the cases re-classified as Unexplained (i.e. SIDS), Undetermined (when the post-mortem was incomplete or incorrectly performed), or Explained (due to illnesses, accidental suffocation or unnatural causes), according to a classification that has recently been proposed in an attempt to standardise the coding criteria at an international level [15]. Globally, 20 audits were carried out, 11 to classify the “grey-zone” cases and 9 to discuss the causes underlying incorrect management of the procedure.

Audits are an important tool for improving joint working practices, preventing future failures in identifying SUID cases, and ensuring appropriate analyses of the cause of death.

Italian mortality data on SUID, recently published in this journal, show a very low incidence: 0.1 and 0.04 ‰ for SUDI and SIDS respectively, accounting for more than half the cases in our region [16]. We have already expressed our concern regarding the reliability of these data [17], because they were obtained from the death certificates and therefore they are not fully compliant with the criteria for a reliable diagnosis. The correct diagnostic approach to every SUID, must necessarily include a thorough post-mortem examination, an investigation of the death scene and a case discussion. In Italy, these three steps are seldom carried out, therefore diagnosis is unfortunately only presumptive in most cases. Due to the difficulty of the diagnostic challenge a complex organisation is required in order to ensure a comprehensive approach to SUID cases. Indeed, different incidence rates are reported in various countries depending on variable coding [18]. Moreover, some sudden infant deaths remain “unexplained” even after a correctly performed post-mortem. Models similar to ours for the management of SUDI are reported in literature [19, 20], and a comparison between these programs allows for identifying the main desirable points listed below for ensuring an efficient approach to the problem:

- careful multi-agency planning of the response
- ongoing consideration of the family’s psychological and emotional needs
- inclusion of referrals for bereavement support
- initial assessment and management, including a detailed and thorough history
- examination of the infant, preliminary medical and forensic investigations
- immediate care of the family, including siblings
- an assessment of the environment and circumstances of the death
- a standardised and thorough post-mortem examination
- a final multi-professional case-discussion meeting.

In our retrospective evaluation we found a satisfactory agreement with most of the items above listed. Some aspects such as the initial communication are crucial for ensuring correct support for bereaved families [21]. Emergency services represent the first contact with the families therefore they play a central role in the grieving process. Based on this consideration, educational meetings before the execution of the protocol were held with members of SIDS Centre and Emergency Services representatives to share information, proposals and experiences. The group drew up a brochure (see Additional file 2) which contains general information on SIDS and recommendations for a correct first communication. The leaflet has been distributed to all emergency departments of our region. For other aspects like the death scene investigation we evaluated our approach as incomplete. This limitation arises because our form contains few items and moreover it is filled in quickly by the emergency staff in critical circumstances. Improvement actions must be undertaken to obtain more comprehensive death-scene investigations which include an extensive array of information regarding the home environment and sleep-related circumstances. The contribution of the death scene investigation to the diagnosis is considered extremely relevant [22], but while on one hand this aspect is important, on the other, its overestimation could be misleading. Indeed, recent data show that in the USA, the diagnosis of SIDS is giving way to coding as accidental suffocation, strangulation, wedging, asphyxia and ill-defined deaths. This diagnostic shift is due to the application of a complex algorithm based on careful data collection obtained through the extensive evaluation of death circumstances [23]. This approach has provoked a heated debate within the “SIDS community” because its uncritical adoption could lead to the abolition of SIDS as a discrete pathological entity [24, 25]. In other words, the “triple risk” model [26] which encompasses the interplay between a window of vulnerability (2–4 months of age), a biological vulnerability (brainstem lesions) and risk factors (prone position during sleep, etc.), to explain SIDS phenomenon, could be abandoned. In our and other researchers’ opinion [27] this approach cannot be fully approved because an increasing amount of data demonstrates that biological vulnerability is part of the tragical pathway leading to SIDS even if, in the large majority of cases, one or more risk factors must be present at the scene to induce the sudden death of an infant considered healthy up until that moment [28–31].

Finally, diagnosis as “undetermined death”, when used to describe a death of unknown origin, may provoke medical legal consequences, and have a negative psychological impact entailing social stigma for bereaved families [32].

## Conclusions

Our structured multiagency approach for SUID ensures care for families and sensitive investigation into the cause of death. The low mortality rate arising from the favourable impact of the ‘Safe to Sleep’ message, encourages us to continue our efforts towards achieving a wider dissemination of prevention strategies, particularly among non-Italian parents for ensuring a greater reduction of sudden infant deaths in the future. Improvement actions are also mandatory for refining death scene investigations because data obtained from our form are too scarce to give an accurate and consistent picture of the circumstances of death. To overcome this limitation, approval of the National Protocol for SUID is necessary, which foresees the in-tandem intervention of a pathologist and a coroner committed to carrying out a professional death scene investigation. The final approval of this act and its execution should allow for abolishing disparities in the management of SUID in Italy, and provide adequate support for bereaved families throughout the country.

## Supplementary information

**Additional file 1.** SUID cases form. This form is filled by the emergency staff. It cointains the data related to the death scene.

**Additional file 2.** Guidebook fot Emergency Staff. The brochure provides general information on SIDS and recommendations for a correct first communication.

## Data Availability

The datasets generated and analysed during the current study are not publicly available but are available from the corresponding author on reasonable request.

## Abbreviations

ALTE: Apparent Life-Threatening Event
SIDS: Sudden Infant Death Syndrome
SIP: Italian Paediatrics Society
SUDI: Sudden Unexpected Deaths in Infancy
SUEND: Sudden Unexpected Early Neonatal Death
SUPC: Sudden Unexpected Postnatal Collapse

## References

[1] Cheng TL, Monteiro N, DiMeglio LA, Chien AT, Peeples ES, Raetz E, Scheindlin B, Denne SC (2016). Seven great achievements in pediatric research in the past 40 y. Pediatr Res.

[2] Willinger M, James LS, Catz C (1991). Defining the sudden infant death syndrome (SIDS): deliberations of an expert panel convened by the National Institute of Child Health and Human Development. Pediatr Pathol.

[3] Sidebotham P, Pearson G (2009). Responding to and learning from childhood deaths. BMJ..

[4] Garstang J, Griffiths F, Sidebotham P (2014). What do bereaved parents want from professionals after the sudden death of their child: a systematic review of the literature. BMC Pediatr.

[5] Tuscany Region. Regional Decree N. 2011 dated 12 08 2008..

[6] Tuscany Region. Regional Decree N. 1164 dated 14-12-2009.

[7] Lavista Ferres JM, Anderson TM, Johnston R, Ramirez JM, Mitchell EA (2020). Distinct Populations of Sudden Unexpected Infant Death Based on Age. Pediatrics.

[8] Becher J-C (2011). Guidelines for the investigation of newborn infants who suffer a Sudden and Unexpected Postnatal Collapse in the First Week of Life Recommendations from a Professional Group on Sudden Unexpected Postnatal Collapse.

[9] Protocollo di indagini e di riscontro diagnostico nella morte improvvisa infantile - Legge 2 febbraio 2006, n. 31, art.1, comma 2″ e "Morte inaspettata di feto di età gestazionale superiore alla 25a settimana") Protocol of investigations and diagnostic findings in Sudden Unexpected Infant Deaths. Law no. 31 of 2 February 2006. art. 1 subsection 2, and “Unexpected death of a foetus after the 25^th^ week of gestational age”.

[10] Rizzo S, De Gaspari M, Carturan E, Paradiso B, Favretto D, Thiene G, Basso C. A standardized postmortem protocol to assess the real burden of sudden infant death syndrome. Virchows Arch. 2020:1–7. 10.1007/s00428-020-02747-2. 10.1007/s00428-020-02747-2 PMC7371652 31975036

[11] Moscovis SM, Hall ST, Burns CJ, Scott RJ, Blackwell CC (2014). The male excess in sudden infant deaths. Innate Immun.

[12] Fleming PJ, Blair PS, Pease A (2015). Sudden unexpected death in infancy: Aetiology, pathophysiology, epidemiology and prevention in 2015. Arch Dis Child.

[13] Blair PS, Sidebotham P, Berry PJ, Evans M, Fleming PJ (2006). Major epidemiological changes in sudden infant death syndrome: a 20-year population-based study in the UK. Lancet..

[14] Centers for Disease Control and Prevention (1990). Seasonality in sudden infant death syndrome — United States, 1980–1987. MMWR Morb Mortal Wkly Rep.

[15] Goldstein RD, Blair PS, Sens MA, Shapiro-Mendoza CK, Krous HF, Moon RY, Rognum TO (2019). Inconsistent classification of unexplained sudden deaths in infants and children hinders surveillance, prevention and research: recommendations from the 3rd international congress on sudden infant and child death. Forensic Sci Med Pathol.

[16] Campi R, Bonati M (2019). Can we still do something-and what?-for a seemingly missing syndrome?. It.J.Ped.

[17] Piumelli R, Arzilli C, Nassi N, Peruzzi M, Ernst C-M, Salvatori C (2019). Can we still do something and what? For a seemingly missing syndrome?: “yes we can”. It JPed.

[18] Taylor BJ, Garstang J, Engelberts A, Obonai T, Cote A, Freemantle J, Vennemann M, Healey M, Sidebotham P, Mitchell EA, Moon RY (2015). International Comparison of Sudden Unexpected Death in Infancy Rates Using a Newly Proposed Set of Cause-Of-Death Codes. Arch Dis Child.

[19] Kennedy H, Bates H, Debelle G, Lishman S (2016). Sudden unexpected death in infancy and childhood. Multi-agency guidelines for care and investigation. The report of a working group convened by the Royal College of pathologists and endorsed by the Royal College of paediatrics and child health.

[20] Goldstein RD, Nields HM, Kinney HC (2017). A New Approach to the Investigation of Sudden Unexpected Death. Pediatrics.

[21] Meert KL, Eggly S, Pollack M (2008). Parents’ perspectives on physician-parent communication near the time of a child’s death in the pediatric intensive care unit. Pediatr Crit Care Med.

[22] Fleming PJ, Blair PS, Sidebotham PD, Hayler T (2004). Investigating Sudden Unexpected Deaths in Infancy and Childhood and Caring for Bereaved Families: An Integrated Multiagency Approach. BMJ.

[23] Shapiro-Mendoza CK, Camperlengo L, Ludvigsen R, Cottengim C, Anderson RN, Andrew T, Covington T, Hauck FR, Kemp J, MacDorman M (2014). Classification system for the sudden unexpected infant death case registry and its application. Pediatrics..

[24] Cutz E (2016). The disappearance of sudden infant death syndrome. JAMA Pediatr.

[25] Schmidt CJ (2016). The disappearance of sudden infant death syndrome. JAMA Pediatr.

[26] Filiano JJ, Kinney HC (1994). A perspective on neuropathologic findings in victims of the sudden infant death syndrome: the triple-risk model. Biol Neonate.

[27] Moon RY, Hauck FR. SIDS Risk: It's More Than Just the Sleep Environment. Pediatrics. 2016;137(1). 10.1542/peds.2015-3665 Epub 2015 Dec 2.PMID: 26634773. 10.1542/peds.2015-3665 26634773

[28] Paterson DS, Trachtenberg FL, Thompson EG, Belliveau RA, Beggs AH, Darnall R, Chadwick AE, Krous HF, Kinney HC (2006). Multiple serotonergic brainstem abnormalities in sudden infant death syndrome. JAMA.

[29] Garcia AJ, Koschnitzky JE, Ramirez J-M (2013). The physiological determinants of sudden infant death syndrome. Respir Physiol Neurobiol.

[30] Randall BB, Paterson DS, Haas EA, Broadbelt KG, Duncan JR, Mena OJ, Krous HF, Trachtenberg FL, Kinney H (2013). Potential asphyxia and brainstem abnormalities in sudden and unexpected death in infants. Pediatrics.

[31] Haynes RL, Frelinger AL, Giles EK, Goldstein RD, Tran H, Kozakewich HP, Haas EA, Gerrits AJ, Mena OJ, Trachtenberg FL, Paterson DS, Berry GT, Adeli K, Kinney HC, Michelson AD (2017). High serum serotonin in sudden infant death syndrome. Proc Natl Acad Sci U S A.

[32] Crandall LG, Reno L, Himes B, Robinson D. The Diagnostic Shift of SIDS to Undetermined: Are There Unintended Consequences?.Acad Forensic Pathol. 2017;7(2):212-220. doi: 10.23907/2017.022 Epub 2017 Jun. 10.23907/2017.022 PMC6474538 31239975

