# Supplementary material for: The multiagency approach to Sudden Unexpected Infant Deaths (SUID): eleven years’ experience in the Tuscany Region
Source: Ital J Pediatr. 2020 Jul 20;46:99. doi: 10.1186/s13052-020-00867-8 (PMC7372863; doi:10.1186/s13052-020-00867-8)
Supplement: Supplementary file 1 — Additional file 1. SUID cases form. This form is filled by the emergency staff. It cointains the data related to the death scene. [file 13052_2020_867_MOESM1_ESM.pdf]

Seeds for SIDS ONLUS

Regional SIDS Centre

Sudden Infant Death Syndrome (SIDS)/ Emergency Staff data collection

Death Scene

Name____________________________ Date of birth ___________________________

found dead on _______________________ at___________ by mother □ father □ others □ specify ___________

death site:

In hospital □ name____

At home: □

in the crib/bed: in parent's room □ in another room □

in another place: □ specify____

bedsharing with parents: No □ Yes □

bedsharing with someone else □ specify_____________________________________

high chair: No □ Yes □ in arms: No □ Yes □

in the stroller: No □ Yes □ in the infant-seat: No □ Yes □

other: □ specify____

Environmental temperature: Too hot □, Too cool □, Normal □

Body temperature: ________ °C

Cigarette smoke: No □ Yes □

Not at home: □ specify______________

Stroller □ car seat □ in arms □

Someone else's home □ specify____

Position: on tummy □ on back □ on side □ □ in another position specify_______________________

Clothing ____

Bedding: Blankets: No □ Yes □ sheets: No □ Yes □

Pillow: No □ Yes □ teddy bear in the baby's bed: No □ Yes □

Other: □ specify________________________________

Pacifier: No □ Yes □

Time of the last meal________________

Biological material:	in the mouth	No □ Yes □ specify__________________________
	in the nose	No □ Yes □specify __________________________
	in the nappy	No □ Yes □ specify __________________________
Hypostatic spots		No □ Yes □
Rigor mortis		No □ Yes □
CPR		No □ Yes □

1. Consider the possibility that the infant has died of SIDS

2. Explain that the death is not attributable to parental carelessness

3. Create a support net around the family (SIDS Centre, Parent's Association)

4. Talk about the opportunity of performing a post-mortem examination

How to alert the Regional SIDS Centre:

1. **Call the Operation Centre on 118 and transmit the data via fax or email**
2. **The Operation Centre will take care of alerting the SIDS Centre**

**Emergency Staff Doctor**	**Family Paediatrician**
**Name**	**Name**
**Surname**	**Surname**
**Phone Number**	**Phone Number**
