# Supplementary material for: The multiagency approach to Sudden Unexpected Infant Deaths (SUID): eleven years’ experience in the Tuscany Region
Source: Ital J Pediatr. 2020 Jul 20;46:99. doi: 10.1186/s13052-020-00867-8 (PMC7372863; doi:10.1186/s13052-020-00867-8)
Supplement: Supplementary file 2 — Additional file 2. Guidebook fot Emergency Staff. The brochure provides general information on SIDS and recommendations for a correct first communication. [file 13052_2020_867_MOESM2_ESM.pdf]

Sudden Infant Death Syndrome (SIDS)

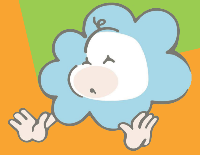

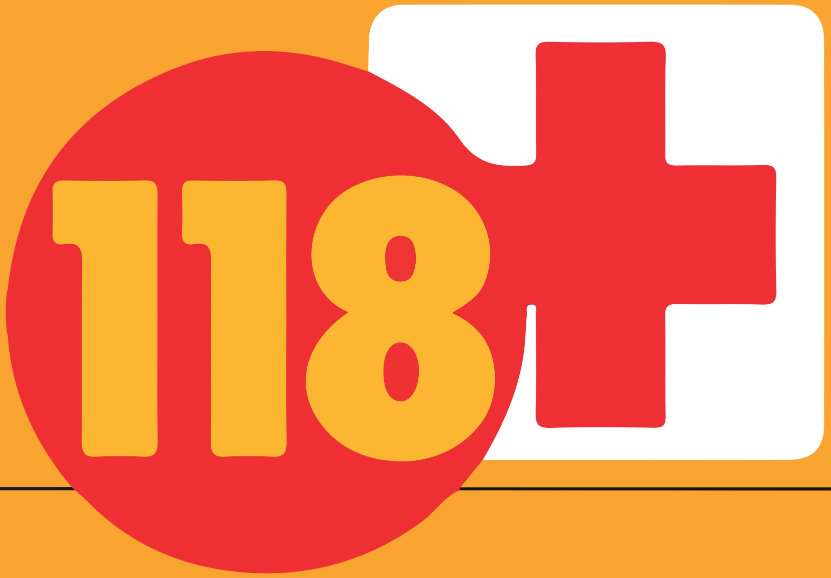

Guidebook for Emergency Staff

Dipartimento di Patologia
Umana e Oncologia
Firenze

Università di Siena
Dipartimento di Patologia
Umana e Oncologia
Sezione di Anatomia
Patologica

U.O. Anatomia Patologica
Universitaria

Azienda Ospedaliero
Universitaria Firenze

Sistema Emergenza-Urgenza
Regione Toscana

Federazione Italiana
Medici Pediatri
Toscana

Associazione
Semi per la SIDS onlus

Italian Association
SEEDS for SIDS

Centro Regionale SIDS

Azienda
Ospedaliero
Universitaria
MEYER

Regione Toscana
Dipartimento della Sanità
e delle Politiche della Salute

This booklet is for the emergency staff who frequently represent the first contact for SIDS families

SIDS occurs above all at home, therefore the emergency staff's involvement is of primary importance.
These healthcare professionals must be properly advised and trained in order to provide a correct "first communication" for the families and collect useful data for an early clinical picture.
Furthermore, the emergency staff should represent a link between families, the Regional SIDS Centre, and the Parents Association in order to provide early support to bereaved families.

What is SIDS?

Sudden Infant Death Syndrome (SIDS), also known as Crib Death, was classified as a separate disease in 1969.
The term SIDS means the sudden and unexpected death of a healthy infant that frequently occurs during sleep and remains unexplained even after a thorough investigation including an autopsy, death-scene investigation, and review of the clinical history.
Less frequently, after undergoing this critical event at home the infant later dies in hospital (delayed SIDS). These events must also be managed as instructed in this booklet.

What causes SIDS?

As implied by the definition, the causes of SIDS are still unknown.
Finding a clinical cause underlying these events has always been difficult.
Initially, a link was assumed between these deaths and overlaying during bed sharing.
Following, SIDS was ascribed to fatal suffocation due to regurgitation or parental carelessness.
Nowadays it is well known that none of these situations are linked to SIDS.
Recent international studies suggest that SIDS could be the result of a multifactorial action, hypothesising that a complex deficiency in respiratory control during sleep may play a crucial role.
This deceptive situation is limited to the very first months of life, but unfortunately this vulnerability cannot be identified with screening tests.
Regardless of the cause, SIDS is never the result of neglect and it can occur in healthy babies with a high level of caregiving. Nor can the typical problems of infants (i.e. fever, abdominal pain, etc.) herald a case of SIDS.

SIDS: the numbers

SIDS is the leading cause of death in infants between 1 month and 1 year of age. It occurs more frequently between the second and fourth month of life and about 60% of the victims are males.
In Italy, no reliable data on the incidence of SIDS are available due to the lack of exhaustive post-mortem examinations.
Conversely, a correct classification and precise epidemiological evaluation of these deaths is extremely important, both for research purposes and for providing early support for bereaved families.

The impact on families

The death of a baby is always devasting for the whole family. If possible, a SIDS death is even more distressing because of the overwhelming sense of guilt.
The unexpected and unexplained nature of these deaths and the deep feelings between the parents and the baby concur to create a shattering sense of chaos and unreality.
For many parents this represents the first experience with death, therefore they are probably totally unprepared for the grief and changes in their emotional, physical and mental balance.

The death scene

During the first examination of a suspected SIDS death it is important to assess the "rigor mortis" and the presence of blood, mucus, slime and vomit near the mouth or nose. Any hypostatic spots on the body could provide information about the infant's position at the time of the death.
Data collected at the death scene are very important both for a clinical diagnosis and research.

The role of the Emergency Services: Why is my baby dead?

The first communication provided by the emergency service is very important because parents will be looking for answers to what has happened and why.
The communication should be empathetic and the emergency staff must carefully:
1: Consider the possibility that the infant has died of SIDS
2. Explain that the death is not attributable to parental carelessness
3. Create a support net around the family (SIDS Centre, Parents Association)
4. Talk about the opportunity of performing a post-mortem examination

Conclusion

SIDS is an event that deeply affects everyone involved.
Because of the multiple reactions, it is crucial to find the correct way to express one's own feelings in order to support the bereaved families.
